# The Discovery of Grape Sugar

**Published:** 1883-03

**Authors:** 


The Discovery of Grape Sugar.—At the present time, glucose, or artificial grape sugar, is being made in such quantities, and so much has been said and written for and against it, some regarding it as identical with the natural sugar of fruits and honey, others as differing from it physiologically, if not chemically, that our readers will be interested in the early history of the substance. The first mention of it was made by T. Lowitz in an article published in Crell's *Chemische Annalen* for the first half of the year 1792. This journal, then one of the leading scientific periodicals, is now so scarce that no public library in New York City is in possession of a set of it.

Lowitz discovered a peculiar kind of sugar in honey, and gives in that journal a quaint but interesting description of the manner in which he discovered and prepared it.

We may conclude, says Lowitz, that honey owes its sweetness to the abundance of sugar that it contains, but that no one knew how to separate this from the other constituents. This separation was the chief object of his experiments. First he succeeded in removing the peculiar taste, odor and color by filtering the diluted honey over charcoal, but on attempting to concentrate the solution by evaporation, it turned brown and showed no tendency to crystallize.

After several months, he continues, there appeared in my honey, which had been treated with charcoal and again concentrated, some very small, white, shining bodies of crystalline nature, which gradually increased in quantity, and finally filled out, for the greater part, the whole mass of honey. In order to be able to investigate the nature of this granular substance, it was necessary to carefully remove from it the brown, thick, sticky substances. This succeeded best by washing it with cold, highly rectified spirits of wine (alcohol). I was delighted to see that, by mixing strongly together, the alcohol dissolved the adhesive matter without perceptibly attacking the white granular particles, which were finally entirely cleansed from it by frequent washing with fresh alcohol. The sugary substance that remained on the filter, after gently drying, could be rubbed to a fine and perfectly white powder, which did not attract moisture from the air and possessed an agreeable sweet taste.

The author goes on to state that all his efforts to obtain regular crystals were fruitless, that the crystalline masses always resembled cauliflowers (or warts), and consisted of very fine needles. He also found that the solution was turned brown by lime water, and after precipitating the lime an acid remained. This sugar was also decomposed by other caustic alkalies, he says. After discussing the difference in the action of this sugar and other sugar toward alkalies, Lowitz adds that the other substance extracted from the honey sugar by alcohol differs from it in no other respect except that it cannot be obtained in a dry form by any means ; that to this latter substance honey owes its property of turning brown by heat, for when the honey sugar has been freed from it the solution can be boiled over the fire without browning. Moreover, this sweet, sticky substance resembles honey sugar in all other properties, as well in taste as in its action toward caustic alkalies and quicklime.

He concludes with the assertion that there is no hope of our ever being able to make sugar from honey, as something more is necessary than to merely remove the foreign substances.

Reading these remarks with the light that ninety years of research have thrown upon them, they stand forth as remarkable instances of correct observation. The crystals which he obtained were dextrose, or ordinary glucose, while the uncrystallized sugar is now known as levulose, a mixture of the two constituting fruit sugar as it exists in honey.

Ten years or more elapsed before Kirchoff discovered the now important fact that this form of sugar could be made from starch by the action of dilute acids; and a half a century rolled away before the manufacture of sugar from starch became one of the large—very large—chemical industries.—*Scientific American.*

---

HOSPITAL SUNDAY DISTRIBUTION.

The Hospital Saturday and Sunday Fund Committee yesterday agreed upon the ratio of distribution of the sums collected through the various channels employed by the association. The report of the committee shows that the total amount of designated and undesignated funds collected was $33,762.72; and this amount, minus $3,935.56, was distributed among the following institutions:

| | |
|---|---|
| St. Luke's Hospital | $7,674 04 |
| Hospital for Ruptured and Crippled | 405 00 |
| Mount Sinai Hospital | 4,200 00 |
| German Hospital | 3,255 00 |
| Presbyterian Hospital | 4,191 03 |
| St. Mary's Hospital | 2,052 01 |
| House of Rest for Consumptives | 1.420 00 |
| House of Holy Comforter | 1,228 75 |
| Home for Incurables | 956 71 |
| New York Eye and Ear Infirmary | 217 60 |
| New York Infirmary for Women and Children | 561 00 |
| New York Ophthalmic Hospital | 570 00 |
| Hahnemann Hospital | 946 02 |
| French Hospital | 555 00 |
| Orthopedic Hospital | 570 00 |
| Ophthalmic and Aural Institute | 550 00 |
| Manhattan Eye and Ear Hospital | 475 00 |
| Total | $29,827 16 |

